# Perivascular vital cells in the ablation center after multibipolar radiofrequency ablation in an in vivo porcine model

**DOI:** 10.1038/s41598-021-93406-2

**Published:** 2021-07-06

**Authors:** F. G. M. Poch, C. A. Neizert, B. Geyer, O. Gemeinhardt, S. M. Niehues, J. L. Vahldiek, K. K. Bressem, K. S. Lehmann

**Affiliations:** 1 Department of General, Visceral and Vascular Surgery, Charité – Universitätsmedizin Berlin, Corporate Member of Freie Universität Berlin, Humboldt-Universität zu Berlin, and Berlin Institute of Health, Campus Benjamin Franklin, Berlin - Hindenburgdamm 30, 12203 Berlin, Germany; 2 Department of Radiology, Charité – Universitätsmedizin Berlin, Corporate Member of Freie Universität Berlin, Humboldt-Universität zu Berlin, and Berlin Institute of Health, Campus Benjamin Franklin, Berlin - Hindenburgdamm 30, 12203 Berlin, Germany

**Keywords:** Medical research, Oncology, Gastrointestinal cancer

## Abstract

Multibipolar radiofrequency ablation (RFA) is an advanced ablation technique for early stage hepatocellular carcinoma and liver metastases. Vessel cooling in multibipolar RFA has not been systematically investigated. The objective of this study was to evaluate the presence of perivascular vital cells within the ablation zone after multibipolar RFA. Multibipolar RFA were performed in domestic pigs in vivo. Three internally cooled bipolar RFA applicators were used simultaneously. Three experimental settings were planned: (1) inter-applicator-distance: 15 mm; (2) inter-applicator-distance: 20 mm; (3) inter-applicator-distance: 20 mm with hepatic inflow occlusion (Pringle maneuver). A vitality staining was used to analyze liver cell vitality around all vessels in the ablation center with a diameter > 0.5 mm histologically. 771 vessels were identified. No vital tissue was seen around 423 out of 429 vessels (98.6%) situated within the central white zone. Vital cells could be observed around major hepatic vessels situated adjacent to the ablation center. Vessel diameter (> 3.0 mm; *p* < 0.05) and low vessel-to-ablation-center distance (< 0.2 mm; *p* < 0.05) were identified as risk factors for incomplete ablation adjacent to hepatic vessels. The vast majority of vessels, which were localized in the clinically relevant white zone, showed no vital perivascular cells, regardless of vessel diameter and vessel type. However, there was a risk of incomplete ablation around major hepatic vessels situated directly within the ablation center. A Pringle maneuver could avoid incomplete ablations.

## Introduction

Radiofrequency ablation (RFA) represents a curative therapy option for early stage hepatocellular carcinoma (HCC) and liver metastases^1–4^. Although RFA is considered to be curative, local recurrence rates between 10 and 40% have been reported for conventional (mono- and bipolar) RFA^5–7^. Tumor size, tumor localization and vicinity to major hepatic vessels are predictive factors for tumor recurrence after thermal ablation^8^. Enhanced RFA systems like multibipolar RFA have been developed in order to overcome these limitations^9^. In multibipolar RFA, several electrodes can be placed around the tumor in dependence of tumor size and the respective RFA system. The distance between the electrodes is referred to as the “no-touch gap”^10^. Larger ablations can be achieved in multibipolar RFA in comparison to conventional RFA^11^. Therefore, an adequate safety margin around the tumor is more likely^11^. Additionally, the risk of tumor seeding along the needle track is reduced, if direct tumor contact can be avoided^12^. Favorable results have been reported for small (< 3 cm), medium (3–5 cm) and even large (> 5 cm) HCC when treated with multibipolar RFA^13–15^.

Complete removal of a liver tumor is determined by a subsequent histopathological analysis of the resected tissue after surgery. In contrast to surgical resection, a histopathological examination is not possible after multibipolar RFA. Instead, technical success is indirectly determined by medical imaging techniques like magnetic resonance imaging (MRI), contrast-enhanced computed tomography (CECT) or contrast enhanced ultrasound (CEUS)^16,17^. An ablation zone can be subdivided into two histopathological ablation zones. The inner white zone represents the area where complete cell destruction occurs^18,19^. This zone merges into the so-called “red zone” which is characterized by the appearance of partially non-vital and vital liver tissue. Although imaging quality has improved over the years, no statement regarding vital tissue immediately adjacent to hepatic vessels directly running through the ablation zone can be made. This seems to be especially important since distinct cooling effects have been reported for hepatic RFA^8^. Minor residual tumor tissue may result in local recurrences.

## Objective

The objective of this study was to evaluate the presence of perivascular vital cells within the thermal ablation zone after multibipolar RFA. A vitality staining was used to analyze liver cell vitality around all vessels with a diameter ≥ 0.5 mm histologically. While vital cells should be expected in the peripheral red zone, no vital cells should be detectable in the central white zone of an ablation^18,19^. We therefore hypothesize that no viable cells remain within the clinically relevant inner white zone.

## Results

**Figure 1:**
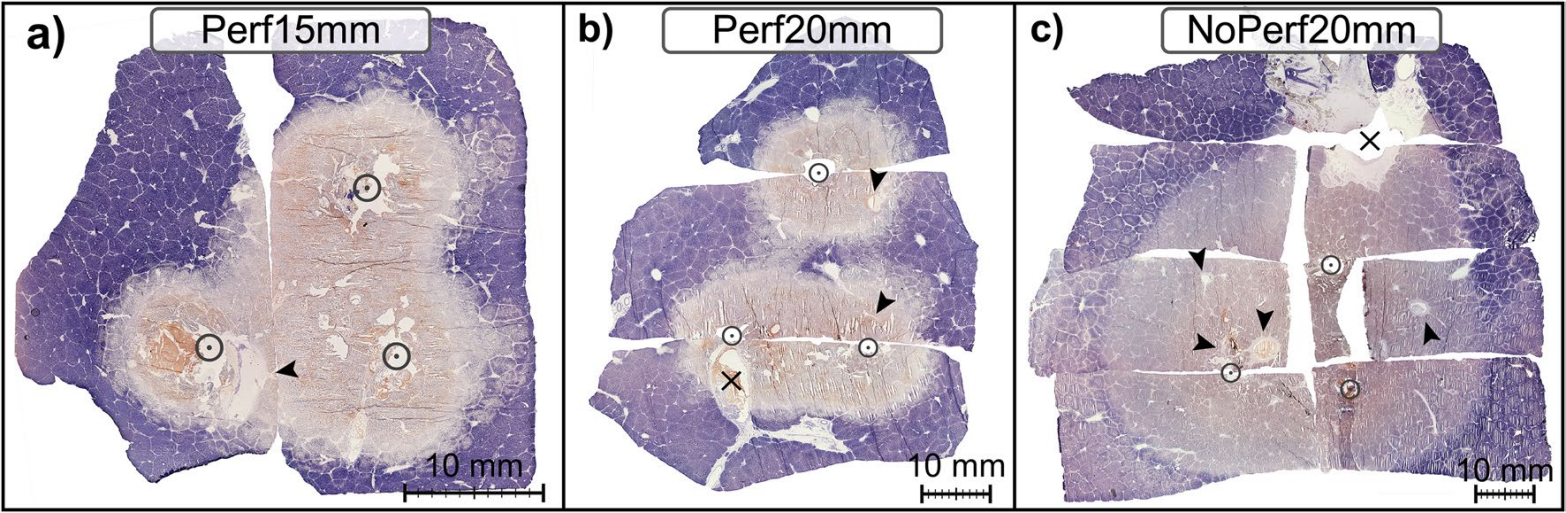
Exemplary display of histological ablation shapes (NADH staining) for each ablation setting with an applicator distance of 15 mm (**a**), 20 mm (**b**) and 20 mm with Pringle maneuver (**c**). All vessels with a diameter ≥ 0.5 mm were analyzed in each histological slide. Vessel type, tissue vitality around each vessel and vessel localization were evaluated. Vessels situated at the border between ablation zones (X) were categorized to the zone which contained its largest areal proportion. (arrowhead = ablated vessels situated in the WZ; circle = applicator puncture site).

A total of 24 multibipolar RFA were performed in 12 domestic pigs [age: 161 days (114–180); weight: 69 kg (62–80)]. One ablation had to be terminated due to cardiovascular instability of an animal during a Pringle maneuver (NoPerf20mm). Therefore, seven ablations for NoPerf20mm and eight ablations for Perf15mm and Perf20mm each were included in the analysis. A preplanned starting power of 90 W (J/s) and a total energy input of 50 kJ were accomplished in all experiments. Irregular shaped ablations occurred in the experimental setting with an applicator distance of 20 mm and sustained hepatic perfusion (Perf20mm, Fig. 1). In this test series 75% of the ablations showed incomplete coagulation at the ablation center. Roundish and confluent ablations were achieved by reducing the applicator distance (Perf15mm) or interrupting hepatic perfusion (NoPerf20mm). A central white zone could be identified in all three experimental settings. No native liver tissue was seen macroscopically within the central white zone adjacent to hepatic vessels.

**Figure 2:**
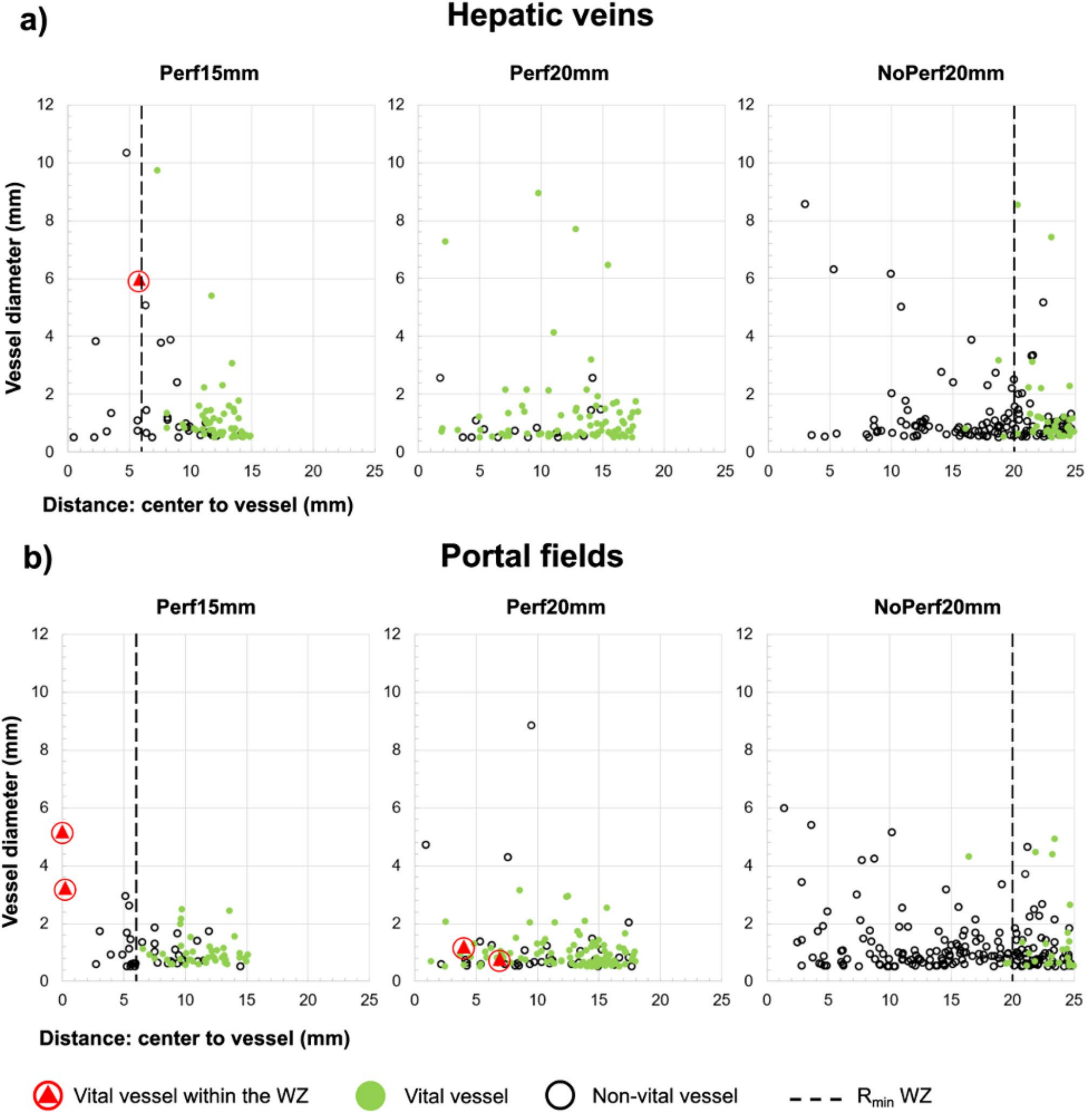
Visualization of all hepatic vessels (n = 771) in dependence of vessel type and experimental setting. Tissue around these vessels was either circularly ablated (black circles) or (partly) vital (green dots). The vertical dashed line shows the median minimal ablation radius (R_min_ white zone) of the respective test series. This line indicates the minimum ablation size. No minimal ablation radius could be shown for Perf20mm, since the ablation center was not ablated in 75% of the cases. Red encircled triangles indicate incomplete ablation adjacent to vessels situated within the WZ. This is of particular importance as no vital cells are expected within this zone.

In total, 771 hepatic vessels were identified in all 23 ablations. Hepatic arteries represented the smallest fraction with 48 vessels (6%), 379 were portal veins (49%) and 344 were hepatic veins (45%). Of all 771 vessels, 429 were situated within the white zone (56%), 125 within the red zone (16%) and 217 within native liver tissue (28%; Fig. 2). A cooling effect of portal veins and portal arteries could not be determined individually due to their anatomical proximity. They were therefore grouped as portal fields. Complete ablation around all vessels within the WZ was possible regardless of vessel type and diameter when a Pringle maneuver was performed (NoPerf20mm). Due to incomplete coagulation of the ablation center, vital vessels could be observed in close proximity to the middle of an ablation (Perf20mm). A reduction of applicator distance resulted in a larger number of ablated hepatic veins and portal fields around the ablation center (Perf15mm).

**Figure 3:**
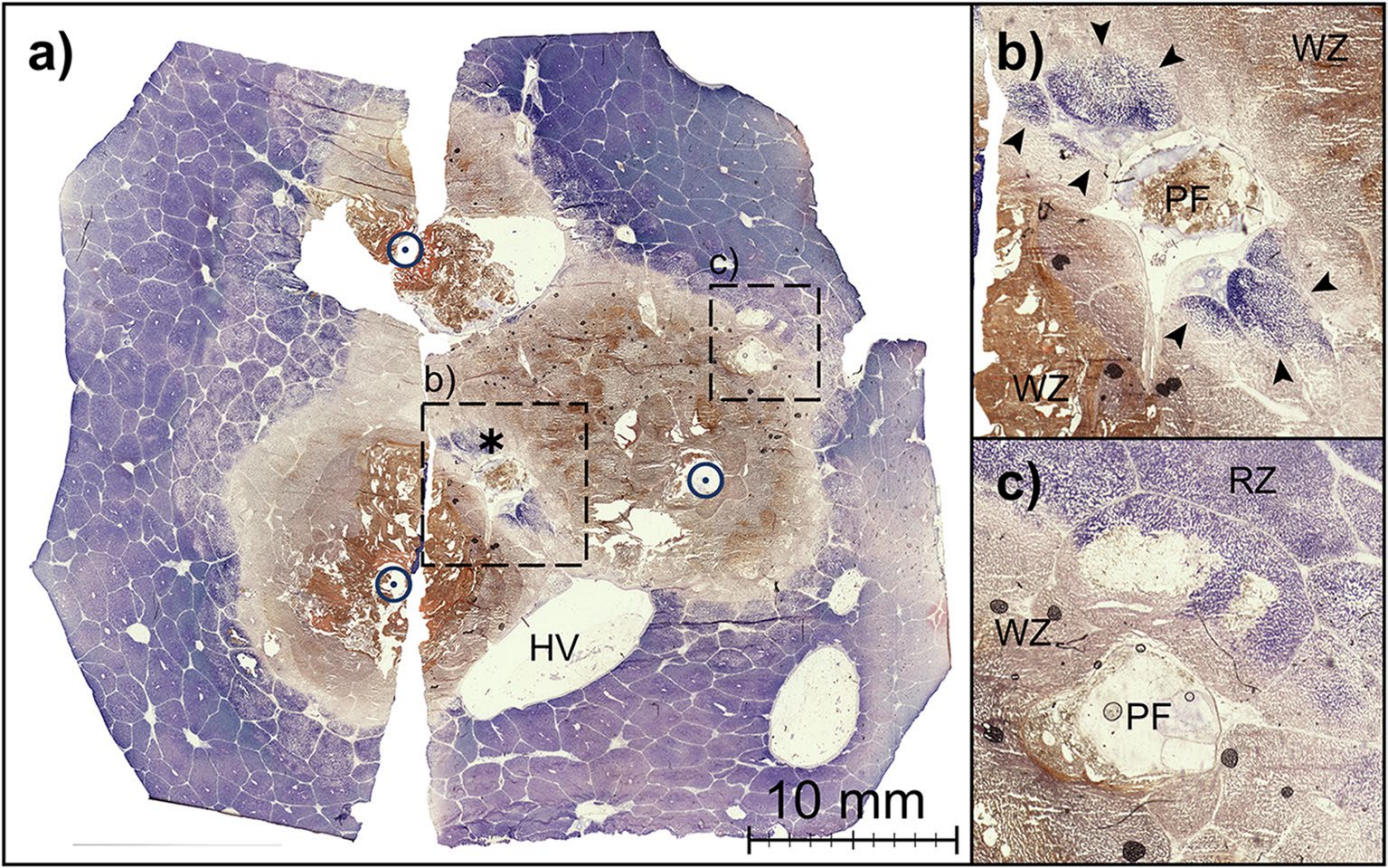
(**a**) Exemplary histological cross-section (NADH staining) of a confluent ablation (Perf15mm) with a partially vital hepatic vessel in the ablation center (asterisk). (**b**) Enlarged view of this vessel (portal field, 3.2 mm in diameter). Vital tissue (Right inverted triangle) as well as partially ablated tissue was observed adjacent to this portal field. (**c**) In contrast to the centrally located portal field, no vital tissue was seen around this portal field (3.0 mm in diameter) in the periphery of the ablation zone. (open circle = applicator puncture site; asterisk = ablation center; PF = portal field; HV = hepatic vein; WZ = white zone; RZ = red zone).

All vessels localized within the red zone showed vital perivascular liver tissue. A precise analysis of all vessels situated within the clinically relevant central WZ is shown in Table 1. The largest ablation area was observed in NoPerf20mm. Therefore, 39.7% (306/771) of all identified vessels were observed in the white zone of this test series. Partially vital liver tissue was histologically identified adjacent to 11 (2.6%) out of 429 vessels in the white zone. Six of these vessels were located at the border of the white zone. In these cases, vital liver tissue surrounding the vessel belonged to the adjoining red zone. All of these borderline vessels showed complete destruction of the liver tissue adjacent to the WZ and were therefore counted as “non-vital” in Table 1. The remaining five vessels showed partial tissue vitality in the clinically relevant WZ (red encircled triangles in Fig. 2). Two of these five vessels were observed in Perf20mm and were situated between the applicators of irregular shaped ablations. The diameter of these vessels was < 2.0 mm. In contrast, three vessels with adjacent vital liver tissue were observed within the white zone center of round and homogeneous ablations (Perf15mm). One of these vessels had a diameter of 6.0 mm and was 5.9 mm away from the ablation center (Perf15mm: Fig. 2a). The other two vessels (portal veins, Perf15mm: Fig. 2b) had a diameter of 3.2 mm and 5.2 mm respectively and were situated exactly in the center of the ablation (center to vessel distance: < 0.2 mm; Fig. 3).

**Table 1:** Histological vitality around all vessels with a diameter of ≥ 0.5 mm situated within the white zone in dependence of vessel diameter and vessel distance to the ablation center^a^.

Vitality	n (%)	Median vessel diameter (mm)		Median distance^a^ (mm)	
**Perf15mm**					
Non-vital	68 (95.8)	0.7 (0.5–6.0)		8.1 (0.5–14.5)	
Part. vital	3 (4.2)	5.2 (3.2–6.0)	*p* = 0.007	0.2 (0–5.9)	*p* = 0.020
**Perf20mm**					
Non-vital	50 (96.2)	0.7 (0.5–8.8)		9.7 (0.9–17.7)	
Part. vital	2 (3.8)	1.0 (0.8–1.2)	*p* = 0.436	5.5 (4.0–6.9)	*p* = 0.151
**NoPerf20mm**					
Non-vital	306 (100)	0.9 (0.5–8.6)		17.4 (1.5–25.3)	
Part. vital	0	–	–	–	–

Due to a larger ablation area compared to the ablation settings with preserved hepatic perfusion, a greater number of vessels (306/424) could be identified within the WZ when a Pringle maneuver was performed (NoPerf20mm).

## Discussion

Our study showed that complete ablation of all hepatic vessels situated within the clinically relevant white zone is possible regardless of vessel diameter, vessel type, liver perfusion and applicator positioning. However, the risk of incomplete ablation increased with uninterrupted physiological blood flow and applicator distances of 15 mm and 20 mm when vessels with a diameter > 3.0 mm were situated close to or directly in the ablation center. In total, 5 out of 429 vessels showed remnant vital tissue within the central white zone. Thus, the ablation center potentially represents the vulnerable point in multibipolar RFA.

The “heat-sink” effect of hepatic vessels is a known risk factor for incomplete RFA^20,21^. Ablations in close vessel vicinity are associated with tumor recurrence rates up to 37% in conventional mono- and bipolar RFA^8^. In comparison, low local tumor recurrence rates have been described for multibipolar RFA, suggesting a reduced susceptibility towards vascular cooling effects^6,22,23^. Larger ablation volumes are achieved due to a higher energy deposition^24^. Clinical studies also demonstrated that HCC and colorectal metastases abutting major hepatic vessels can be effectively treated with multibipolar RFA^25,26^. However, systematic in situ analyses of vascular cooling effects in multibipolar RFA have not been performed so far. This seems relevant, since an ex vivo study showed that distinct vascular cooling effects do exist in multibipolar RFA. These cooling effects were independent of applicator to vessel distance and had a major impact on ablation shape^27^. Furthermore, a numerical study on no-touch bipolar RFA suggested that the central region may not sufficiently be affected by energy supply^10^. Tissue destruction adjacent to the applicator is induced actively by joule heating. In contrast, heating in the periphery of the ablations takes place passively by thermal conduction. Ablations are more susceptible to vascular cooling effects in the periphery of an ablation^28^. This effect may be the cause for incomplete ablations due to insufficiently overlapping ablation zones in the center of multibipolar RFA. Distinct cooling effects caused by the vessels in the ablation periphery were observed in our study. These cooling effects resulted in a reduced ablation area compared to the test settings with Pringle maneuver. It was not possible to assign these cooling effects to individual vessels due to the large number of present. Instead, it seems necessary to confirm the technical ablation success by means of postinterventional imaging in clinical practice, as cooling effects are difficult to predict prior RFA^29^. Our study directs attention to centrally located larger vessels around which vital cells may persist. Vital cells around these vessels cannot be identified by medical imaging and therefore pose a hidden risk of tumor recurrence. For this reason, a Pringle maneuver should be considered in such cases to safely avoid potentially residual tumor cells in this area. In conventional RFA, vessel diameter seems to be a critical factor for accomplishing ablation success. Lu et al. determined a vessel diameter threshold of 3 mm above which the heat sink effect was observed consistently^20^. A comparable threshold does not yet exist for multibipolar RFA. We could demonstrate in our in vivo study, that within round and confluent ablations, vital cells around centrally located vessels with a diameter of > 3.0 mm could persist. This may lead to incomplete ablations. Besides vessel diameter, studies indicate that vessel type plays a vital role in thermal ablations^30–32^. So far, there are no systematic in vivo studies which have evaluated the cooling effect directly adjacent to specific hepatic vessels in multibipolar RFA histologically. We could show that incomplete ablation occurred only around 5 out of 429 vessels within the WZ. Four of these partially vital vessels were portal fields. This number of vessels is too small to draw any definitive conclusions. However, it indicates that stronger cooling effects occur around portal fields in comparison to hepatic veins in multibipolar RFA. This is in accordance with other studies of RFA, laser induced thermotherapy (LITT) and MWA^30–32^. Frericks et al. not only attribute this finding to higher blood flow velocities and profiles but also to a more complex microvascular branching pattern of portal fields in relation to hepatic veins^32^. It is uncertain which of these mechanisms is more relevant. However, ex vivo studies imply that flow velocities ≥ 1 ml/min do not affect the size of the heat sink effect significantly^33,34^. Thus, further research is necessary to avoid incomplete tumor ablation adjacent to major portal fields in clinical practice.

Several studies suggest the use of a Pringle maneuver to potentiate perivascular cell death around major hepatic vessels^20,35^. We could show that a Pringle maneuver resulted in complete ablation of all vessels independent of their diameter and type within the ablation center. However, clamping of the hepatoduodenal ligament requires at least a laparoscopic intervention. Moreover, there is a considerable risk of ischemic liver damage^36,37^.

A limitation of our study is the use of native porcine liver. However, domestic pigs are acknowledged as an experimental model due to comparable anatomical and physiological properties to human liver tissue. Currently, there is no tumor model for swine. Porcine liver is rather flat so that we used applicators with an active length of 30 mm to achieve complete ablations. Typically, active applicator lengths of 40 mm are used in clinical practice. It seems likely that larger applicator lengths prevent wide gaps in between the applicators. Another limitation is that the analysis was performed immediately after an ablation. Our study is able to confirm technical success but is incapable to certify therapeutic sufficiency over the course of time. Vital tissue detected adjacent to major hepatic vessel in the ablation center may perish over time. Long-term studies are desirable to confirm our results.

Multibipolar RFA is a promising option for patients with early stage HCC and liver metastases^4,13,14^. However, round and confluent ablations with a sufficient safety margin > 5 mm are needed for an adequate treatment of hepatic malignancies^38^. As previously reported by our research group, round and homogenous ablations are possible with a Pringle maneuver, while irregular ablations are likely when the applicator distance is too large during physiological blood flow in multibipolar RFA^39^. Knowledge of the effects of hepatic vessels on ablation success is crucial to further improve ablation planning. This is especially relevant as ablation success can only be indirectly evaluated through modern imaging techniques such as CECT and MRI^12,29^. Nevertheless, even modern postinterventional medical imaging is unable to display possible vital tissue in the ablation center. Our present study shows that incomplete ablation between the applicators appears if vessels > 3.0 mm are situated directly in the WZ. Hence, ablation success is not only dependent on applicator distancing but also the presence of major hepatic vessels in close vicinity to the ablation center when there is continuous blood flow.

In conclusion, the ablation center seems to be the vulnerable spot in multibipolar RFA. A Pringle maneuver should be considered in clinical routine when large hepatic vessels are located in immediate tumor proximity.

## Methods

### Workflow overview

Experiments were conducted under general anesthesia in domestic pigs. Multibipolar RFA was performed in the opened abdominal cavity under CT-guidance. Three different ablations settings were planned. The animals were euthanized before liver explantation. The ablations were cut into half and frozen immediately afterwards. A vitality staining was used after preparing histological samples. All hepatic vessels with a diameter ≥ 0.5 mm were identified and histologically analyzed. Details on the individual steps of the workflow are given below.

### Animals

An in vivo porcine model was used in this study. Domestic pigs were chosen since the liver size of these animals is suitable for multibipolar RFA. Experiments were performed in native liver tissue as no adequate tumor model is available. All experiments were approved by the regional health authority (Landesamt für Gesundheit und Soziales Berlin, Germany; application number: G0281/12). Statutory regulations were followed according to the German law and European guidelines on animal welfare (2010/63/EU, FELASA). Details of the administration of anesthetics and the operative procedures have been described before^18,29^. The authors complied with the ARRIVE guidelines.

### Multibipolar RFA

Multibipolar RFA was performed with an impedance controlled RFA generator (CelonSurgical, Olympus Surgical Technologies Europe, Hamburg, Germany). Three internally cooled bipolar RFA applicators (CelonProSurge 150-T30, Olympus Surgical Technologies Europe, Hamburg, Germany; active length: 30 mm; diameter: 1.8 mm) were used simultaneously in our study. Two of the six electrodes were alternately activated for two seconds by the RF-generator. The generator switched between fifteen possible electrode combinations according to a standardized protocol. Starting power was set to 90 W pursuant to the manufacturer’s recommendations. Ablations were manually stopped when energy input reached 50 kJ. The three applicators were placed into the liver manually in a parallel position and triangular configuration. A spacer ensured exact distancing between the applicators. Contrast-enhanced computed tomography (“CECT”; Aquilion PRIME, Canon Medical Systems) was performed as final assessment of the applicator position using a multi-phase protocol after intravenous contrast medium application (100 ml Imeron 400 MCT, Bracco Imaging Deutschland GmbH, Konstanz, Germany). CECT was carried out before ablation to reposition the applicators in the liver to avoid proximity to the liver surface or contact with neighboring organs. Only after the desired applicator position was confirmed by CECT, ablation was started. Three different ablation settings (each n = 8) were planned:

1. Perf15mm: 15 mm applicator distance and obtained natural liver perfusion
2. Perf20mm: 20 mm applicator distance and obtained natural liver perfusion
3. NoPerf20mm: 20 mm applicator distance and hepatic inflow occlusion (Pringle maneuver)

After RFA and a subsequent hepatectomy, ablations were cut along the maximum cross-sectional diameter. The maximum diameter was defined by the insulator situated between the two electrodes at the tip of each applicator.

### Evaluation of intrahepatic vessels

Ablations were subdivided into squares of 25 × 45 × 10 mm to fit microscopic slides. These squares were frozen immediately with liquid nitrogen and embedded in TissueTek (Sakura Color Products Corporation, Osaka, Japan). A cryostat (CryoStar NX70, Thermo Fisher Scientific Inc., Waltham, USA) was used to achieve histological samples (6–8 µm). A vitality staining was carried out according to Neumann et al.^40^. Histological slides were digitalized with a scanner (Nikon SupercoolScan 5000 ED, Nikon Corporation, Tokyo, Japan).

All hepatic vessels with diameters of ≥ 0.5 mm were identified in each ablation. The distance of each vessel to the ablation center was measured. In addition, vessel type (hepatic vein, portal vein or hepatic artery), vessel localization (white zone, red zone or native liver tissue) and histological vitality around the vessel were recorded. Vessels were classified into three different categories:

- Vital: the vessel is completely enclosed by vital tissue
- Non-vital: the vessel is completely enclosed by non-vital tissue
- Partially vital: the vessel is enclosed by vital as well as non-vital tissue

A vessel situated at the border to another ablation zone was categorized to the zone which contained its largest areal proportion.

### Statistical analysis

Statistical analysis was conducted with a statistical software (IBM SPSS Statistics 25, International Business Machines Corporation, New York, USA, https://www.ibm.com/analytics/spss-statistics-software). Categorical variables are displayed as count (percentage). Continuous variables were tested with the Shapiro–Wilk test for normal distribution, which was not given. Data are therefore expressed as median (minimum–maximum). The Mann–Whitney-U-test was used to compare two independent groups. The level of significance was 0.05 (two sided) for each statistical testing.

## Data Availability

The datasets analysed in our study are available from the corresponding author on reasonable request.

## References

[1] Llovet JM, Burroughs A, Bruix J (2003). Hepatocellular carcinoma. Lancet.

[2] Bruix J, Reig M, Sherman M (2016). Evidence-based diagnosis, staging, and treatment of patients with hepatocellular carcinoma. Gastroenterology.

[3] Izzo F (2019). Radiofrequency ablation and microwave ablation in liver tumors: An update. Oncologist.

[4] Gruber-Rouh T (2016). Current strategies in interventional oncology of colorectal liver metastases. Br. J. Radiol..

[5] Gao J (2015). Radiofrequency ablation for single hepatocellular carcinoma 3 cm or less as first-line treatment. World J. Gastroenterol..

[6] Chai Y, Li K, Zhang C, Chen S, Ma K (2019). The short-term efficacy of no-touch radiofrequency ablation in treating cirrhosis-based small hepatocellular carcinoma. BMC Cancer.

[7] Vitali GC (2016). Minimally invasive surgery versus percutaneous radio frequency ablation for the treatment of single small (≤ 3 cm) hepatocellular carcinoma: A case-control study. Surg. Endosc..

[8] Mulier S (2005). Local recurrence after hepatic radiofrequency coagulation: Multivariate meta-analysis and review of contributing factors. Ann. Surg..

[9] Frericks BB, Ritz JP, Roggan A, Wolf KJ, Albrecht T (2005). Multipolar radiofrequency ablation of hepatic tumors: Initial experience. Radiology.

[10] Yap, S. *et al.* A numerical study on the no-touch bipolar radiofrequency ablation. In: *Conference Proceedings IEEE Engineering in Medicine and Biology Society* 2019, 2887–2890. 10.1109/EMBC.2019.8857816 (2019). 10.1109/EMBC.2019.8857816 31946494

[11] Seror O (2014). Histopathologic comparison of monopolar versus no-touch multipolar radiofrequency ablation to treat hepatocellular carcinoma within Milan criteria. J. Vasc. Interv. Radiol..

[12] Kawamura Y (2019). No-touch ablation in hepatocellular carcinoma has the potential to prevent intrasubsegmental recurrence to the same degree as surgical resection. Hepatol. Res..

[13] Mohkam K (2018). No-touch multibipolar radiofrequency ablation vs. surgical resection for solitary hepatocellular carcinoma ranging from 2 to 5cm. J. Hepatol..

[14] N'Kontchou G (2019). Multibipolar radiofrequency ablation for the treatment of mass-forming and infiltrative hepatocellular carcinomas > 5 cm: Long-term results. Liver Cancer.

[15] Petit A (2020). No-touch multi-bipolar radiofrequency ablation for the treatment of subcapsular hepatocellular carcinoma ≤ 5 cm not puncturable via the non-tumorous liver parenchyma. Cardiovasc. Intervent. Radiol..

[16] Goldberg SN, Gazelle GS, Mueller PR (2000). Thermal ablation therapy for focal malignancy: A unified approach to underlying principles, techniques, and diagnostic imaging guidance. AJR Am. J. Roentgenol..

[17] Bouda D (2016). Imaging review of hepatocellular carcinoma after thermal ablation: The good, the bad, and the ugly. J. Magn. Reson. Imaging.

[18] Gemeinhardt O (2016). Comparison of bipolar radiofrequency ablation zones in an in vivo porcine model: Correlation of histology and gross pathological findings. Clin. Hemorheol. Microcirc..

[19] Mulier S (2007). Experimental and clinical radiofrequency ablation: Proposal for standardized description of coagulation size and geometry. Ann. Surg. Oncol..

[20] Lu DS (2003). Influence of large peritumoral vessels on outcome of radiofrequency ablation of liver tumors. J. Vasc. Interv. Radiol..

[21] Goldberg SN (1998). Percutaneous radiofrequency tissue ablation: Does perfusion-mediated tissue cooling limit coagulation necrosis?. J. Vasc. Interv. Radiol..

[22] Seror O (2016). Hepatocellular carcinoma within Milan criteria: No-touch multibipolar radiofrequency ablation for treatment-long-term results. Radiology.

[23] Hocquelet A (2017). Comparison of no-touch multi-bipolar vs. monopolar radiofrequency ablation for small HCC. J. Hepatol..

[24] Seror O (2016). Hepatocellular carcinoma within Milan criteria: No-touch multibipolar radiofrequency ablation for treatment-long-term results. Radiology.

[25] Snoeren N (2015). Multipolar radiofrequency ablation for colorectal liver metastases close to major hepatic vessels. Surgeon.

[26] Loriaud A (2018). Hepatocellular carcinoma abutting large vessels: Comparison of four percutaneous ablation systems. Int. J. Hyperth..

[27] Poch FG (2016). The vascular cooling effect in hepatic multipolar radiofrequency ablation leads to incomplete ablation ex vivo. Int. J. Hyperth..

[28] Schramm W, Yang D, Wood BJ, Rattay F, Haemmerich D (2007). Contribution of direct heating, thermal conduction and perfusion during radiofrequency and microwave ablation. Open Biomed. Eng. J..

[29] Vahldiek JL (2018). Multipolar RFA of the liver: Influence of intrahepatic vessels on ablation zones and appropriateness of CECT in detecting ablation dimensions—Results of an in-vivo porcine liver model. Clin. Hemorheol. Microcirc..

[30] Bangard C (2006). Experimental radiofrequency ablation near the portal and the hepatic veins in pigs: Differences in efficacy of a monopolar ablation system. J. Surg. Res..

[31] Poch FG (2020). Periportal fields cause stronger cooling effects than veins in hepatic microwave ablation: An in vivo porcine study. Acta Radiol..

[32] Frericks BB (2008). Influence of intrahepatic vessels on volume and shape of percutaneous thermal ablation zones: In vivo evaluation in a porcine model. Invest. Radiol..

[33] Lehmann KS (2009). Ex situ quantification of the cooling effect of liver vessels on radiofrequency ablation. Langenbecks Arch. Surg..

[34] Lehmann KS (2016). Minimal vascular flows cause strong heat sink effects in hepatic radiofrequency ablation ex vivo. J. Hepatobiliary Pancreat. Sci..

[35] Rhaiem R (2020). Microwave thermoablation of colorectal liver metastases close to large hepatic vessels under pringle maneuver minimizes the "heat sink effect". World J. Surg..

[36] Ypsilantis P (2011). Pringle maneuver exacerbates systemic inflammatory response and multiple-organ injury induced by extended liver radiofrequency ablation. Hum. Exp. Toxicol..

[37] van Riel WG, van Golen RF, Reiniers MJ, Heger M, van Gulik TM (2016). How much ischemia can the liver tolerate during resection?. Hepatobiliary Surg. Nutr..

[38] Hirooka M (2019). Prospective cohort trial to confirm the efficacy of no-touch radio frequency ablation. J. Gastroenterol. Hepatol..

[39] Poch FGM (2020). Influence of interapplicator distance on multibipolar radiofrequency ablation during physiological and interrupted liver perfusion in an in vivo porcine model. Sci. Rep..

[40] Neumann RA, Knobler RM, Leonhartsberger H, Bohler-Sommeregger K, Gebhart W (1991). Histochemical evaluation of the coagulation depth after argon laser impact on a port-wine stain. Lasers Surg. Med..

